# Facility Fees for Colonoscopy Procedures at Hospitals and Ambulatory Surgery Centers

**DOI:** 10.1001/jamahealthforum.2023.4025

**Published:** 2023-12-15

**Authors:** Yang Wang, Yuchen Wang, Elizabeth Plummer, Michael E. Chernew, Gerard Anderson, Ge Bai

**Affiliations:** 1 Department of Health Policy and Management, Johns Hopkins Bloomberg School of Public Health, Baltimore, Maryland; 2 Neeley School of Business, Texas Christian University, Fort Worth; 3 Burnett School of Medicine, Texas Christian University, Fort Worth; 4 Harvard Medical School, Boston, Massachusetts; 5 Medicare Payment Advisory Commission, Washington, DC; 6 Johns Hopkins University School of Medicine, Baltimore, Maryland; 7 Johns Hopkins Carey Business School, Washington, DC

## Abstract

This cross-sectional study investigates commercial facility fee differences for colonoscopy procedures between US hospitals and ambulatory surgery centers located within the same county and contracting with the same insurers.

## Introduction

Variation in facility fees paid for similar health services across different sites of care has received attention. For example, Medicare pays more for services delivered in hospital outpatient departments than ambulatory surgery centers (ASCs). This has led to recommendations and proposed legislation to equalize payments for some services.^1^ While Medicare-related facility fee differences are well known and Medicare and commercial plans might be concordant, less conclusive evidence exists about variations in the commercial market.^2^ We used new Transparency in Coverage (TIC) data disclosed pursuant to recent regulations requiring health insurers to publicly disclose commercial negotiated rates for specific procedures and facilities^3^ to investigate site-related facility fee differences in the commercial market. We examined within-county, within-insurer commercial facility fee differences between hospitals and ASCs for colonoscopy procedures, which are shoppable, largely homogeneous, and commonly performed in both settings.^4^

## Methods

This cross-sectional study followed the STROBE reporting guideline and used TIC insurer-disclosed pricing data for May 2023 compiled by Turquoise Health. We focused on in-network commercial fee-for-service facility fees disclosed by 4 major health insurers—Anthem, Inc; Cigna Group; Healthcare Service Corporation (HCSC); and UnitedHealthcare—for 3 common colonoscopy procedures (*Current Procedural Terminology* [*CPT*] codes 45378, 45380, and 45385). For each procedure, a facility fee was obtained for every unique combination of insurer, hospital or ASC (identified by national provider identifier), and fee type (negotiated or fee schedule). Median facility fee was used when a combination contained multiple facility fees across plans operated by the insurer. We excluded facility fees expressed as percentages and the 1% highest and lowest values for each procedure as potential data anomalies according to literature using price transparency data.^5^ Institutional review board approval was not sought per 45 CFR §46, because no human participants were involved.

For each procedure, nationwide mean commercial facility fees were compared between hospitals and ASCs using 2-sided *t* tests. To check TIC data validity, we compared the results with mean colonoscopy facility fees for hospitals and ASCs from the 2021 Merative Marketscan research database, which contains commercial claims but not insurer, facility, or county identifiers. Regression models including insurer, negotiated type, and county fixed effects estimated the difference in log-transformed facility fees between hospitals and ASCs located in the same county and contracting with the same insurer. Analysis used Stata, version 17.0. Two-sided *P* < .05 was significant.

## Results

The sample included 13 287 colonoscopy commercial facility fees from 3582 hospitals and 17 052 facility fees from 3899 ASCs located in 50 states and Washington, DC. These were disclosed by Anthem (6955 [22.9%]), Cigna (7862 [25.9%]), HCSC (3606 [11.9%]), and UnitedHealthcare (11 916 [39.3%]).

**Figure 1.**
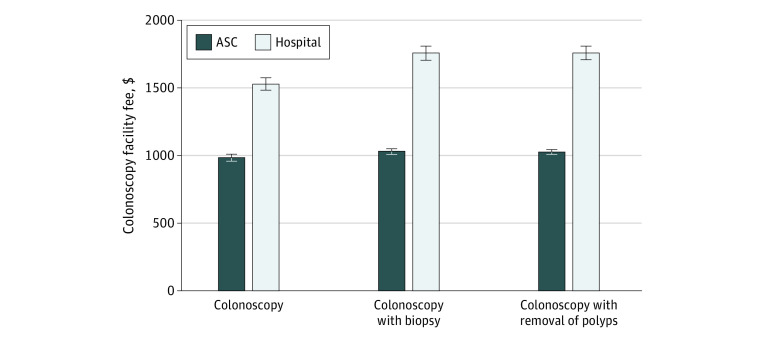
Nationwide Mean Commercial Colonoscopy Facility Fees in Ambulatory Surgery Centers (ASCs) vs Hospitals For colonoscopy, the *Current Procedural Terminology* (*CPT*) code was 45378; for colonoscopy with biopsy, 45380; and for colonoscopy with removal of polyps, 45385. As a validity check, using the 2021 Merative Marketscan research database, we found that the national mean facility fees (unadjusted for inflation) for ASCs and hospitals were $910 vs $1602 for *CPT* code 45378, $897 vs $1709 for *CPT* code 45380, and $900 vs $1674 for *CPT* code 45385. Whiskers indicate 95% CIs.

**Figure 2.**
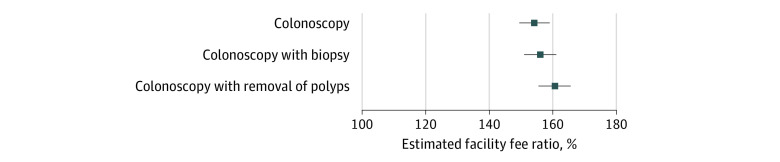
Regression Estimates of Facility Fees for Commercial Colonoscopy Procedures in Hospitals Compared With Ambulatory Surgery Centers For colonoscopy, the *Current Procedural Terminology* (*CPT*) code was 45378; for colonoscopy with biopsy, 45380; and for colonoscopy with removal of polyps, 45385. According to stratified models by insurer, the estimated facility fee ratios were 334%, 367%, and 362% (*P* < .001) for Anthem; 141%, 123%, and 152% (*P* < .001) for Cigna; 165%, 159%, and 160% (*P* < .001) for Healthcare Service Corporation; and 104%, 105%, and 108% (*P* < .001) for UnitedHealthcare for *CPT* codes 45378, 45380, and 45385, respectively. Whiskers indicate 95% CIs.

Nationwide mean facility fees for hospitals ($1530 [95% CI, $1485-$1576], $1760 [95% CI, $1706-$1813], and $1761 [95% CI, $1709-$1814]) were substantially higher than those for ASCs ($989 [95% CI, $970-$1008], $1034 [95% CI, $1015-$1054], and $1030 [95% CI, $1011-$1049]) for CPT codes 45378, 45380, and 45385, respectively (all *P* < .001) (Figure 1). Marketscan 2021 data showed similar fees. After controlling for insurer, negotiated type, and county fixed effects, estimated facility fees in hospitals were 154% (95% CI, 149%-159%), 156% (95% CI, 151%-161%), and 161% (95% CI, 155%-166%) of those in ASCs for *CPT* codes 45378, 45380, and 45385, respectively (all *P* < .001) (Figure 2).

## Discussion

Facility fees at hospitals were approximately 55% higher than those at ASCs in the same county and with the same insurer. Potential limitations involve use of insurers’ self-disclosed pricing information, including use of nonstandard codes, reporting of prices for facilities that do not perform colonoscopies, and no utilization information.^5,6^ Results might not be generalizable to other procedures or nonmajor insurers. Due to data limitations, we did not adjust for variation on system affiliation, case mix, utilization, or quality of care across hospitals or ASCs. Nevertheless, the results suggest that a site-neutral payment policy for a largely homogeneous and shoppable service may generate savings for commercial plan sponsors and beneficiaries.

## Notes

Supplement.
Data Sharing Statement
