# Supplementary material for: Facility Fees for Colonoscopy Procedures at Hospitals and Ambulatory Surgery Centers
Source: JAMA Health Forum. 2023 Dec 15;4(12):e234025. doi: 10.1001/jamahealthforum.2023.4025 (PMC10724760; doi:10.1001/jamahealthforum.2023.4025)
Supplement: Supplement. — Data Sharing Statement [file jamahealthforum-e234025-s001.pdf]

Data Sharing Statement

Wang. Facility Fees for Colonoscopy Procedures at Hospitals and Ambulatory Surgery Centers. *JAMA Health Forum*. Published December 15, 2023.
doi:10.1001/jamahealthforum.2023.4025

Data
Data available: No

Additional Information
Explanation for why data not available: The data used in this study is publicly available.
